# Two-Dimensional Wavenumber Analysis Implemented in Ultrasonic Vector Doppler Method with Focused Transmit Beams

**DOI:** 10.3390/s22249787

**Published:** 2022-12-13

**Authors:** Hideyuki Hasegawa, Masaaki Omura, Ryo Nagaoka, Kozue Saito

**Affiliations:** 1 Faculty of Engineering, University of Toyama, Toyama 930-8555, Japan; 2 Department of Neurology, Stroke Center, Nara Medical University, Nara 634-8522, Japan

**Keywords:** blood flow measurement, autocorrelation technique, vector Doppler, beam steering angle, center frequency

## Abstract

The multi-angle Doppler method was introduced for the estimation of velocity vectors by measuring axial velocities from multiple directions. We have recently reported that the autocorrelation-based velocity vector estimation could be ameliorated significantly by estimating the wavenumbers in two dimensions. Since two-dimensional wavenumber estimation requires a snapshot of an ultrasonic field, the method was first implemented in plane wave imaging. Although plane wave imaging is predominantly useful for examining blood flows at an extremely high temporal resolution, it was reported that the contrast in a B-mode image obtained with a few plane wave emissions was lower than that obtained with focused beams. In this study, the two-dimensional wavenumber analysis was first implemented in a framework with focused transmit beams. The simulations showed that the proposed method achieved an accuracy in velocity estimation comparable to that of the method with plane wave imaging. Furthermore, the performances of the methods implemented in focused beam and plane wave imaging were compared by measuring human common carotid arteries in vivo. Image contrasts were analyzed in normal and clutter-filtered B-mode images. The method with focused beam imaging achieved a better contrast in normal B-mode imaging, and similar velocity magnitudes and angles were obtained by both the methods with focused beam and plane wave imaging. In contrast, the method with plane wave imaging gave a better contrast in a clutter-filtered B-mode image and smaller variances in velocity magnitudes than those with focused beams.

## 1. Introduction

Blood flow measurement is an important practice in clinical diagnoses using ultrasonography. Color flow imaging based on the autocorrelation method [1] is a fundamental element of the function embedded in every clinical scanner and is used to map the Doppler velocities, which are the flow velocity components along the direction of ultrasonic propagation. Although a method was developed for estimation of flow-velocity vectors using Doppler velocities obtained by color flow imaging [2], the method requires assumptions based on the theory of fluid dynamics. Therefore, methods for the direct estimation of flow velocity vectors have been investigated for decades. The speckle tracking method is one of the angle-independent motion estimators and is used to estimate flow velocities [3,4]. The speckle tracking method is particularly useful in cardiac flow imaging where an ultrasonic probe with a small physical aperture must be used [5,6,7,8].

In blood flow imaging of human common carotid arteries, the physical aperture size relative to the imaging depth is significantly larger than that in cardiac imaging. In such cases, the velocity vectors can be estimated by measuring the Doppler velocities from different directions [9,10,11,12,13,14,15]. These methods insonify ultrasonic beams from different directions for measurement of the Doppler velocities. Another strategy for estimation of lateral velocities was developed by Jensen and Munk, where the beamformed ultrasonic signal was modulated in the transverse direction by overlapping two receiving (Rx) beams [16]. The phase of the modulated field provides the transverse velocity of the target.

A high temporal resolution is preferable in blood flow imaging because a blood flow is a highly dynamic phenomenon. An extremely high frame rate of above a thousand frames per second is achieved by plane wave (PW) imaging [17], and the effectiveness of PW imaging in blood flow measurements was demonstrated [18,19]. The coherent plane wave compound (CPWC) was introduced to control the balance between the frame rate and image quality [20], and color flow imaging was realized at a high frame rate with the CPWC technique [21]. The angle-independent motion estimators were also combined with the CPWC technique. In the CPWC sequence, beamformed ultrasonic signals obtained from multiple PW emissions to different directions are coherently compounded. Therefore, velocity vectors can be estimated from the Doppler velocities obtained at different PW steering angles. In such methods, the velocity components along the direction of ultrasonic propagation are estimated by the spectral Doppler [22,23,24], autocorrelation [25,26,27], and speckle tracking [28,29,30]. Other angle-independent motion estimators, such as the transverse oscillation approach [31,32,33] and the frequency domain approach [34,35], were also implemented in imaging schemes with unfocused transmit (Tx) beams, which realized high frame rates.

As described above, high-frame-rate imaging is a promising method for the evaluation of blood flows. On the other hand, it is reported that contrast in a B-mode image obtained with a few plane wave emissions is lower than that obtained with focused Tx beams [20]. Another strategy to realize a high frame rate is a method called multi-line acquisition, where focused Tx beams were transmitted in multiple directions or positions simultaneously [36,37,38]. The multi-line acquisition was applied to myocardial motion imaging [39,40]. Recently, we have implemented the vector Doppler method in the multi-line acquisition scheme for the angle independent estimation of flow velocity in a common carotid artery [41]. However, the variance of the estimated velocities was large due to the lack of beam steering in emission.

In [41], the autocorrelation method was used to estimate the Doppler velocities. The Tx center frequency is commonly used in the autocorrelation method [27], while the Rx center frequency is required. It was shown that the errors in the axial velocity estimates were reduced using the center frequency obtained from the received signal [42,43,44,45,46] and the accuracy of the PW-based vector Doppler method was ameliorated using the estimated center frequency [47]. In addition, the benefit of the center frequency estimation was confirmed in the vector Doppler method with focused beam (FB) imaging [41]. Furthermore, it was recently reported that the velocity estimates obtained with the vector Doppler technique based on autocorrelation were improved further using both the center frequency and tilt angle of the wavefront obtained by estimating the wavenumbers of the beamformed ultrasonic signal in two dimensions [48]. Since the estimation of the wavenumbers requires a snapshot of the ultrasonic field, the method was first developed for PW imaging. In this study, the method presented in [48] was first implemented in the multi-line acquisition scheme with focused Tx beams. In [41], two parallel dynamically focused beams were created in reception to increase the frame rate. In this study, it was shown that such parallel receive beamforming was also required to estimate the two-dimensional wavenumbers. It was also shown theoretically that the total transmit-receive steering angle was halved from the steering angle in reception when the transmit beam was not steered. A small steering angle leads to large errors in estimated velocities [41]. This study demonstrated that the velocity estimation errors under such a small total steering angle could also be suppressed significantly by estimating the two-dimensional wavenumbers for the first time. The fundamental performance of the proposed method with the FB framework was validated by simulations with Field II [49,50]. Then, the performances of the methods implemented in the FB and PW imaging were compared by measuring human common carotid arteries in vivo, and the benefits and shortcomings of both the strategies were discussed.

## 2. Materials and Methods

### 2.1. Tx-Rx Sequence and Beamforming

**Figure 1:**
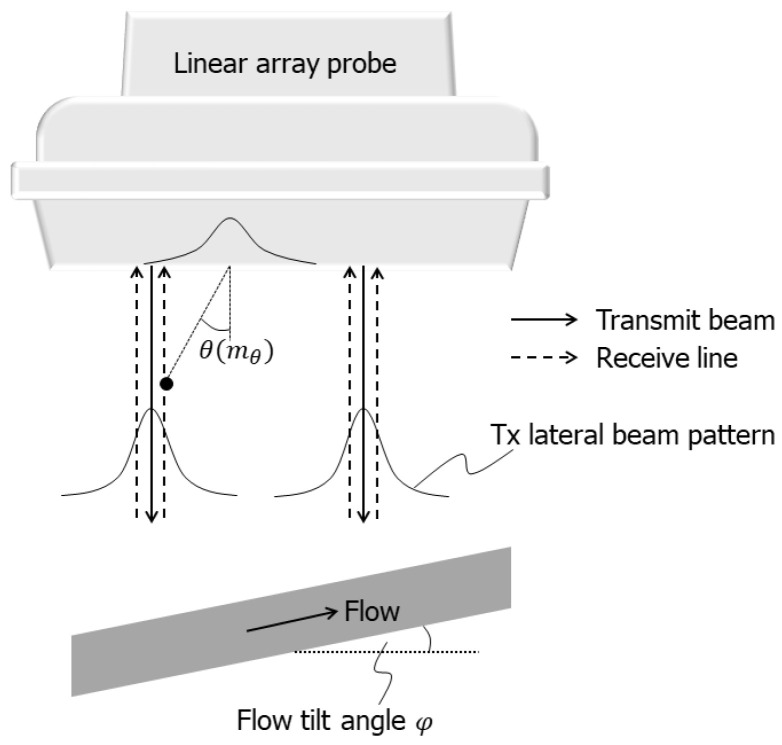
Illustration of measurement geometry and definitions of flow tilt angle and beam steering angle.

As illustrated in Figure 1, two non-steered focused Tx beams were created simultaneously to increase the frame rate. The size of the Tx aperture for each beam was empirically determined as 36 elements (element pitch: 0.2 mm), and Tukey apodization was used at a coefficient of 0.4. The two parallel Tx beams were laterally apart from each other by 60 times the element pitch. In each Tx-Rx event, echo signals were acquired with all the elements in the linear array. As described in the subsequent section, it is necessary to create multiple Rx beams in parallel to estimate the wavenumbers in two dimensions. Therefore, two parallel Rx lines were created around each Tx beam. The lateral interval of the two parallel Rx lines corresponded to the element pitch. The set of the aperture positions for the two parallel Tx beams was laterally translated by two elements after each Tx-Rx event so that the lateral intervals δx of Rx lines always corresponded to the element pitch. By acquiring echo signals at 30 different sets of the aperture positions, (30 sets)×(2 Tx beams)×(2 Rx lines) =120 Rx lines were created. The vertical intervals δz of the sampled beamformed signals in the Rx beamforming were set at 0.025 mm empirically.

The complex analytic signals obtained from the echoes received with the individual elements were used for the Rx delay-and-sum (DAS) beamforming, and the dynamic focusing was performed. Quadrature detection was performed before beamforming to obtain the complex analytic signals because it was necessary to change the beamforming grid depending on the Rx beam steering angle so that the sampled data exist along the axial direction when quadrature detection was performed after Rx beamforming. To avoid such a complexity, the echo signals were converted into the analytic signals before Rx beamforming in this study.

At every sampled point in each Rx line, three Rx beams were created at different steering angles $\theta \left(m_{\theta }\right)\;\left(m_{\theta }=1,\;2,\;\ldots ,\;M_{\theta };\;M_{\theta }=3\right).$ Maximum Rx beam steering angle $\theta_{\max}$ was assigned at 10, 15, and 20 degrees, which corresponded to steering angle sets of (−10, 0, 10), (−15, 0, 15), and (−20, 0, 20) degrees, respectively. To create the Rx beams at the respective steering angles, the peak position of the Rx apodization function (Gaussian function) was set so that the angle from the vertical direction to the direction of the vector from the peak position of the Rx apodization function to the point of interest became the corresponding steering angle as illustrated in Figure 1. The F-number was set at 2.34 in the Rx beamforming, where the full width at half maximum of the Gaussian apodization function was defined as the aperture size.

**Figure 2:**
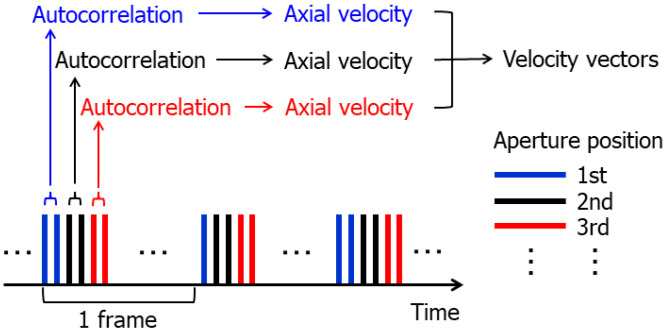
Illustration of transmit sequence. Tx-Rx event was repeated twice at the same aperture position, and autocorrelation was calculated using beamformed signals obtained from two consecutive emissions.

In this study, the multi-line acquisition was performed with the repeated transmit sequence [51]. Figure 2 shows the Tx sequence used in this study. As illustrated in Figure 2, the aliasing limit, i.e., the maximum measurable velocity, remains as large as possible by correlating complex beamformed signals obtained by consecutive emissions.

### 2.2. Principles for Velocity Estimation

In this study, the velocity vector estimator described in [48] was implemented in the multi-line acquisition sequence. The complex beamformed RF signal in each frame, which consists of $M_{x}$ and $M_{z}$ sampled points in lateral and vertical directions, respectively, is defined as $s\left(m_{x},\;m_{z},\;m_{\theta },\;m_{e}\right)\;\left(m_{x}=1,\;2,\;\ldots ,\;M_{x};\;m_{z}=1,\;2,\;\ldots ,\;M_{z};\;m_{\theta }=1,\;2,\;\ldots ,\;M_{\theta };\;m_{e}=1\;\mathrm{or}\;2\right).$ The pulse repetition number at each aperture position is denoted as $m_{e}$.

The autocorrelation function $\gamma \left(m_{x},\;m_{z},\;m_{\theta }\right)$ at a temporal lag is obtained from the complex beamformed signals as follows:

(1) $$\gamma \left(m_{x},\;m_{z},\;m_{\theta }\right)=E_{R}\left[s^{*}\left(m_{x},\;m_{z},\;m_{\theta },\;1\right)\cdot s\left(m_{x},\;m_{z},\;m_{\theta },\;2\right)\right],\;$$

where * denotes complex conjugate, and averaging operation is denoted by $E_{R}\left[\cdot \right]$. In this study, the lateral and vertical sizes of a kernel, which was denoted as R in Equation (1), were set at 1.0 and 0.825 mm, respectively. The Doppler velocity $v_{d}\left(m_{x},\;m_{z},\;m_{\theta }\right)$ is estimated as

(2) $${\hat{v}}_{d}\left(m_{x},\;m_{z},\;m_{\theta }\right)=\frac{c_{0}}{4\pi f_{0}T_{\mathrm{PRI}}}∠\gamma \left(m_{x},\;m_{z},\;m_{\theta }\right),\;$$

where $c_{0},$ $f_{0},$ ∠, and $T_{\mathrm{PRI}}$ denote the speed of sound, ultrasonic center frequency, phase angle of a complex value, and pulse repetition interval (PRI), respectively.

As described in Equation (2), the center frequency $f_{0}$ of the received signal is required to estimate the Doppler velocity. In this study, the method presented in [48] was used to obtain the center frequency $f_{0}\;$ by estimating the wavenumbers of the beamformed signal in two dimensions.

The axial wavenumber k and center frequency $f_{0}$ are estimated as

(3) $${\hat{f}}_{0}=\frac{c_{0}}{2\pi }\hat{k}=\frac{c_{0}}{2\pi }\sqrt{{\hat{k}}_{x}^{2}+{\hat{k}}_{z}^{2}},\;$$

where $k_{x}$ and $k_{z}$ are the lateral and vertical wavenumbers, respectively, and ^ denotes an estimate. The tilt angle $\phi \left(m_{x},\;m_{z},\;m_{\theta }\right)$ of the wavefront in the beamformed signal is also estimated as

(4) $$\hat{\phi }\left(m_{x},\;m_{z},\;m_{\theta }\right)=\tan^{-1}\frac{{\hat{k}}_{x}}{{\hat{k}}_{z}}.\;$$

To estimate the vertical wavenumber $k_{z}$, the autocorrelation function $\gamma_{z}\left(m_{x},\;m_{z},\;m_{\theta }\right)$ at a vertical lag is expressed as

(5) $$\gamma_{z}\left(m_{x},\;m_{z},\;m_{\theta }\right)=E_{R}\left[s^{*}\left(m_{x},\;m_{z},\;m_{\theta },\;1\right)\cdot s\left(m_{x},\;m_{z}+1,\;m_{\theta },\;1\right)\right].\;$$

The vertical wavenumber $k_{z}$ can be estimated from the change in the phase of the beamformed signal during sampling interval δz in the vertical direction, which corresponds to the phase of the autocorrelation function $\gamma_{z}\left(m_{x},\;m_{z},\;m_{\theta }\right)$ at a vertical lag. Thus, the vertical wavenumber $k_{z}$ is estimated as

(6) $${\hat{k}}_{z}=\frac{∠\gamma_{z}\left(m_{x},\;m_{z},\;m_{\theta }\right)}{2\delta z}.\;$$

Similarly, the lateral wavenumber $k_{x}$ is estimated by evaluating the autocorrelation function $\gamma_{x}\left(m_{x},\;m_{z},\;m_{\theta }\right)$ at a lateral lag defined as

(7) $$\gamma_{x}\left(m_{x},\;m_{z},\;m_{\theta }\right)=E_{R}\left[s^{*}\left(m_{x}^{\prime },\;m_{z},\;m_{\theta },\;1\right)\cdot s\left(m_{x}^{\prime }+1,\;m_{z},\;m_{\theta },\;1\right)\right],\;$$

(8) $$m_{x}^{\prime }=\lfloor \frac{m_{x}-1}{2}\rfloor +1,\;$$

where ⌊·⌋ denotes omission of fractions. In the estimation of the lateral wavenumber $k_{x},$ it is important to evaluate the autocorrelation function of the beamformed signals obtained from the same Tx beam because the phase of the autocorrelation function $\gamma_{x}\left(m_{x},\;m_{z},\;m_{\theta }\right)$ includes the phase alterations induced by both the tilt of the wavefront and the target motion when it is evaluated from the beamformed signals obtained from different Tx beams. Therefore, the lateral sampling number $m_{x}^{\prime }$ in Equation (7) was determined by Equation (8) so that the autocorrelation function $\gamma_{x}\left(m_{x},\;m_{z},\;m_{\theta }\right)$ was calculated using the beamformed signals obtained from the same Tx beam (two parallel Rx beams were created in the RX beamforming). The lateral wavenumber $k_{x}$ is estimated as

(9) $${\hat{k}}_{x}=\frac{∠\gamma_{x}\left(m_{x},\;m_{z},\;m_{\theta }\right)}{2\delta x}.\;$$

As described in [9,27], the lateral and vertical velocities $v_{x}\left(m_{x},\;m_{z}\right)$ and $v_{z}\left(m_{x},\;m_{z}\right)$ in each frame are described as

(10) $$v_{x}\left(m_{x},\;m_{z}\right)\left\{\sin\theta_{t}\left(m_{\theta }\right)+\sin\theta_{r}\left(m_{\theta }\right)\right\}+v_{z}\left(m_{x},\;m_{z}\right)\left\{\cos\theta_{t}\left(m_{\theta }\right)+\cos\theta_{r}\left(m_{\theta }\right)\right\}=2v_{d}\left(m,\;m_{z},\;m_{\theta }\right),\;$$

where $\theta_{t}\left(m_{\theta }\right)$ and $\theta_{r}\left(m_{\theta }\right)$ are the Tx and Rx beam steering angles, respectively. A matrix form of the relationships obtained at $M_{\theta }$ different steering angles is expressed as

(11) $$\begin{array}{c} \left[\begin{array}{cc} \begin{array}{c} \sin\theta_{t}\left(1\right)+\sin\theta_{r}\left(1\right)\\ \vdots \\ \sin\theta_{t}\left(M_{\theta }\right)+\sin\theta_{r}\left(M_{\theta }\right) \end{array} & \begin{array}{c} \cos\theta_{t}\left(1\right)+\cos\theta_{r}\left(1\right)\\ \vdots \\ \cos\theta_{t}\left(M_{\theta }\right)+\cos\theta_{r}\left(M_{\theta }\right) \end{array} \end{array}\right]\\ \times \left[\begin{array}{c} v_{x}\left(m_{x},\;m_{z}\right)\\ v_{z}\left(m_{x},\;m_{z}\right) \end{array}\right]=\left[\begin{array}{c} 2{\hat{v}}_{d}\left(m_{x},\;m_{z},\;1\right)\\ \vdots \\ 2{\hat{v}}_{d}\left(m_{x},\;m_{z},\;M_{\theta }\right) \end{array}\right]\\ \Rightarrow Av={\hat{v}}_{\mathrm{ax}}.\; \end{array}$$

The solution of Equation (11) is obtained by the least-square method as

(12) $$\hat{v}={\left(A^{T}A\right)}^{-1}A^{T}{\hat{v}}_{\mathrm{ax}},\;$$

where ${\left(A^{T}A\right)}^{-1}A^{T}$ corresponds to the pseudoinverse of A.

### 2.3. Consideration on Beam Steering Angle in Estimation of Axial Velocity

**Figure 3:**
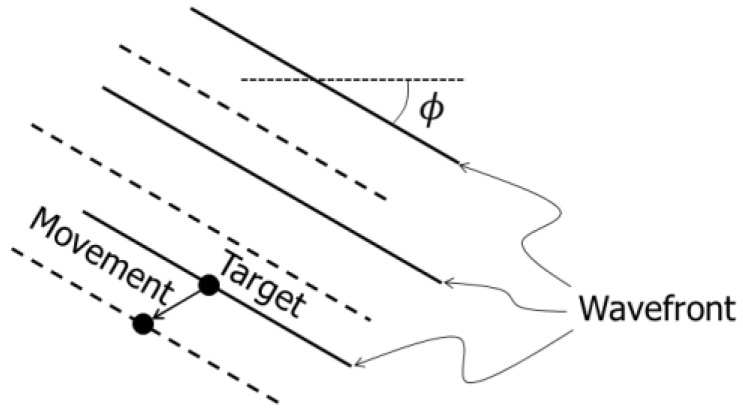
Illustration of target movement in beamformed signal with tilted wavefront. The solid and dashed lines show pre- and post-motion wavefronts, respectively.

As described in Equation (11), the Tx and Rx beam steering angles $\theta_{t}\left(m_{\theta }\right)$ and $\theta_{r}\left(m_{\theta }\right)$ are required to estimate the velocity vector. In the vector Doppler method, the predefined steering angles are commonly used [27,41,47]. This means that $\theta_{t}\left(m_{\theta }\right)=0$ and $\theta_{r}\left(m_{\theta }\right)=\theta \left(m_{\theta }\right)$ in the proposed FB framework. However, the tilt angle of the wavefront in the beamformed signal should be considered because the phase shift of the beamformed signal depends on the tilt angle of the wavefront, as illustrated in Figure 3. The tilt angle of the wavefront in the beamformed signal simply corresponds to the beam steering angle when the Tx and Rx steering angles are the same, as is investigated in [48]. On the other hand, the Tx and Rx steering angles are different in this study. Let us consider the tilt angle of the wavefront in such a situation.

By assuming that the wavefront is locally planar, the ultrasonic field g(x, z) produced from a point target in a Tx or Rx event is simply modelled as

(13) $$g\left(x,\;z\right)=\exp\left(-\frac{x^{2}}{\sigma_{\mathrm{tr}}^{2}}-\frac{z^{2}}{\sigma_{\mathrm{ax}}^{2}}\right)\cdot \exp\left\{j\left(k_{x}x+k_{z}z\right)\right\},\;$$

where $\sigma_{\mathrm{tr}}^{2}$ and $\sigma_{\mathrm{ax}}^{2}$ determine the spread of the Gaussian envelope of the field in the direction of the wavefront and the direction transverse to the wavefront, which depend on the beam width and pulse length, respectively. Although the Gaussian envelope should also be tilted in the case of a steered beam, the tilt of the envelope was omitted because the direction of the wavefront was important in this study.

Since the Tx and Rx beam steering angles are 0 and $\theta \left(m_{\theta }\right)$, respectively, the ultrasonic fields $g_{t}\left(x,\;z\right)$ and $g_{r}\left(x,\;z\right)$ in Tx and Rx are respectively expressed as

(14) $$g_{t}\left(x,\;z\right)=\exp\left(-\frac{x^{2}}{\sigma_{\mathrm{tr}}^{2}}-\frac{z^{2}}{\sigma_{\mathrm{ax}}^{2}}\right)\cdot \exp\left(jkz\right),\;$$

(15) $$g_{r}\left(x,\;z\right)=\exp\left(-\frac{x^{2}}{\sigma_{\mathrm{tr}}^{2}}-\frac{z^{2}}{\sigma_{\mathrm{ax}}^{2}}\right)\times \exp\left[jk\left\{x\sin\theta \left(m_{\theta }\right)+z\cos\theta \left(m_{\theta }\right)\right\}\right].\;$$

With the far field approximation, the Tx-Rx field $g_{t-r}\left(x,\;z\right)$ is expressed by the product of $g_{t}\left(x,\;z\right)$ and $g_{r}\left(x,\;z\right)$ as [52]

(16) $$g_{t-r}\left(x,\;z\right)=\exp\left(-\frac{2x^{2}}{\sigma_{\mathrm{tr}}^{2}}-\frac{2z^{2}}{\sigma_{\mathrm{ax}}^{2}}\right)\times \exp\left[jk\left\{x\sin\theta \left(m_{\theta }\right)+z\left(1+\cos\theta \left(m_{\theta }\right)\right)\right\}\right].\;$$

Therefore, the tilt angle ϕ of the wavefront in the beamformed signal (pulse-echo field) is obtained as

(17) $$\phi =\tan^{-1}\left\{\frac{\sin\theta \left(m_{\theta }\right)}{1+\cos\theta \left(m_{\theta }\right)}\right\}=\frac{\theta \left(m_{\theta }\right)}{2}.\;$$

As can be seen in Equation (17), the tilt angle of the wavefront is different from the predetermined beam steering angle (zero or $\theta \left(m_{\theta }\right))$. As illustrated in Figure 3, the target motion induces the phase shift along the direction transverse to the wavefront in the Tx-Rx field and thus the direction of the estimated Doppler velocity corresponds to this direction. Therefore, in the estimation of velocity vectors in Equation (12), the beam steering angles were set at $\theta_{t}\left(m_{\theta }\right)=\theta_{r}\left(m_{\theta }\right)=\hat{\phi }\left(m_{x},\;m_{z},\;m_{\theta }\right),$ where the tilt angle $\hat{\phi }\left(m_{x},\;m_{z},\;m_{\theta }\right)$ of the wavefront was directly estimated from the beamformed signal using Equation (4).

### 2.4. Clutter Filtering

Clutter filtering based on singular value decomposition (SVD) was performed on the beamformed signals to suppress strong echoes from surrounding tissues and enhance weak echoes from blood cells [41,53]. In the clutter filtering, the beamformed signals obtained with the same steering angle and emission number were grouped for the SVD processing to create datasets with a regular frame interval. In other words, the beamformed signals obtained with different steering angles and emission numbers were processed by SVD separately. In each SVD processing, all 180 frames (approximately 1 s) were processed at once. By inspecting the singular value profiles, the threshold values were assigned empirically to indicate echoes from tissues and blood cells and electrical noise.

### 2.5. Numerical Simulations

The accuracy of the proposed velocity estimator was validated by Field II simulations [49,50]. A 192-element linear array probe was simulated. The element pitch was set at 0.2 mm. Each element emits an ultrasonic pulse of a 3-cycle sinusoidal wave at 4.8 MHz. A Hanning function was used for its envelope. The transmit sequence described in Section 2.1 was implemented, and the echoes from the simulated phantom were received by all the 192 elements. The received signals were stored at a sampling frequency of 31.25 MHz. The pulse repetition frequency (PRF) was set at 10 kHz.

The numerical phantom was generated by distributing point scatterers randomly in a cylindrical shape of 5 mm in diameter. Since a tilt angle of a common carotid artery is generally less than 10 degrees, the flow tilt angle φ of the cylindrical phantom was set at 0, 5, and 10 degrees. The scatterer number density was set at 30.3 scatterers/mm^3^ [24], and the attenuation coefficient was set at 0.5 dB/cm/MHz. The distributed scatterers were moved at a constant speed along the longitudinal direction of the cylindrical shape. Parabolic velocity profiles were assigned, where the moving velocities of the scatterers at the radial periphery of the cylindrical shape were set at zero. The moving velocity was maximum at the center of the transverse section of the phantom. Maximum velocity $v_{\max}$ was altered from 100 to 1000 mm/s.

### 2.6. Experimental Setup

The feasibility of the proposed method in in vivo imaging was demonstrated by measurements of human common carotid arteries of two healthy subjects. This study was approved by the institutional ethical committee and was performed with the informed consent of the subjects.

A custom-made scanner (RSYS0016, Microsonic, Tokyo, Japan) with a 192-element linear array probe (UST-5412, Fujifilm, Tokyo, Japan) was used for in vivo measurements. The element pitch of the linear array was 0.2 mm. The echo signals from the carotid arteries were acquired with the Tx sequence described in Section 2.1. Each element was excited with a rectangular pulse at a center frequency of 4.8 MHz, and the PRI was set at 96 ms, which corresponded to a PRF of 10,417 kHz. The echo signals were received by all the 192 elements and sampled at 31.25 MHz. The settings in the simulations and in vivo measurements are summarized in Table 1.

### 2.7. Metrics for Evaluation of Accuracy in Velocity Estimation

In the simulations, the absolute bias error (ABE) and root mean squared error (RMSE) were used for evaluation of the accuracy in velocity estimation. The ABE and RMSE are defined as

(18) $$ABE=\frac{\left|E_{R_{f}}\left[\hat{v}-v_{\mathrm{tru}}\right]\right|}{\left|E_{R_{f}}\left[v_{\mathrm{tru}}\right]\right|},\;$$

(19) $$RMSE=\frac{\sqrt{E_{R_{f}}\left[{\left|\hat{v}-v_{\mathrm{tru}}\right|}^{2}\right]}}{\left|E_{R_{f}}\left[v_{\mathrm{tru}}\right]\right|},\;$$

where $R_{f}$ and $v_{\mathrm{tru}}$ denote the region with the flow phantom and true velocity vector, respectively. In the simulations, the three methods described below were examined to evaluate the effects of the center frequency and steering angle separately:

1. Method 1: w/o $f_{0}$ estimation, $\theta_{t}\left(m_{\theta }\right)=0$, $\theta_{r}\left(m_{\theta }\right)=\theta \left(m_{\theta }\right)$
2. Method 2: w/$f_{0}$ estimation by Equation (3), $\theta_{t}\left(m_{\theta }\right)=0,$ $\theta_{r}\left(m_{\theta }\right)=\theta \left(m_{\theta }\right)$
3. Method 3: w/$f_{0}$ estimation by Equation (3), $\theta_{t}\left(m_{\theta }\right)=\theta_{r}\left(m_{\theta }\right)=\hat{\phi }\left(m_{x},\;m_{z},\;m_{\theta }\right)$ from Equation (4)

In Method 1, a constant value of 4.3 MHz was assigned as the center frequency over the entire region of interest by referring to ones estimated from the simulated data using Equation (3). Since the steered beams are used in the vector Doppler method, the fully beamformed region is narrowed due to the finite size of the linear array when the steering angle is increased. Therefore, the errors were evaluated within a lateral width of ±4.5 mm around the center of the field of view [48].

## 3. Results

### 3.1. Simulations

#### 3.1.1. Estimation of Tilt Angle of Wavefront

**Figure 4:**
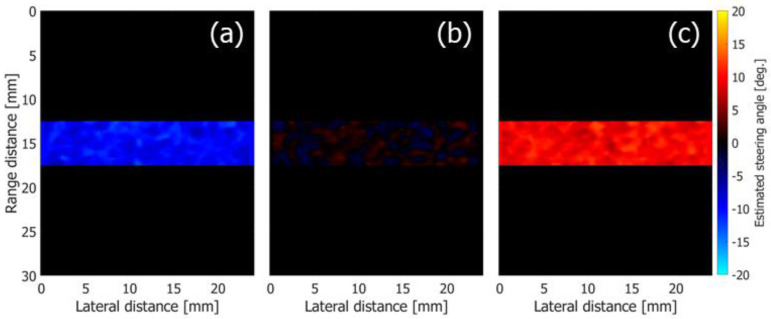
Estimated tilt angles of wavefronts obtained at respective Rx steering angles. (**a**) −20 degrees. (**b**) 0 degrees. (**c**) 20 degrees.

As described in Section 2.3, the tilt angle of the wavefront in the beamformed signal would be different from the Tx or Rx beam steering angle when the Tx and Rx steering angles are different. Therefore, the tilt angles of the wavefronts were first examined at different maximum Rx beam steering angles $\theta_{\max}$. Figure 4a–c show the estimated tilt angles of the wavefronts in the beamformed signal under Rx steering angles of −20, 0, and 20 degrees, respectively. The flow tilt angle φ was set at 0 degrees.

**Figure 5:**
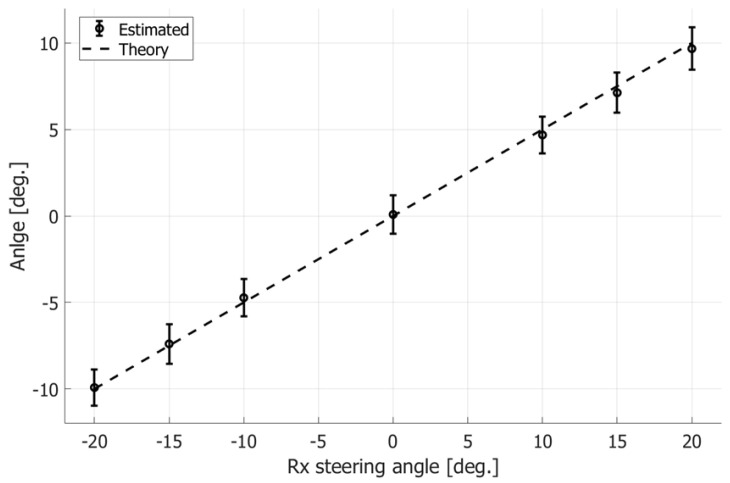
Estimated and theoretical tilt angles of wavefronts obtained at different Rx steering angles.

Although the Rx beam steering angles in Figure 4a,c are set at −20 and 20 degrees, respectively, the estimated tilt angles are different from the preassigned Rx steering angles as described in Section 2.3. The means and standard deviations of the estimated tilt angles of the wavefront in the beamformed signals were obtained at the different Rx beam steering angles and are shown by the plots and vertical bars in Figure 5, respectively. The dashed line shows the theoretical value obtained by Equation (17). Although the absolute values of the estimated tilt angles are slightly lower than the theoretical values, the estimated values are in good agreement with the theoretical ones. As described in Section 2.3, the estimated tilt angle $\hat{\phi }\left(m_{x},\;m_{z},\;m_{\theta }\right)$ of the wavefront was used in the proposed method (Method 3) for estimation of velocity vectors.

#### 3.1.2. Accuracy in Velocity Estimation

**Figure 6:**
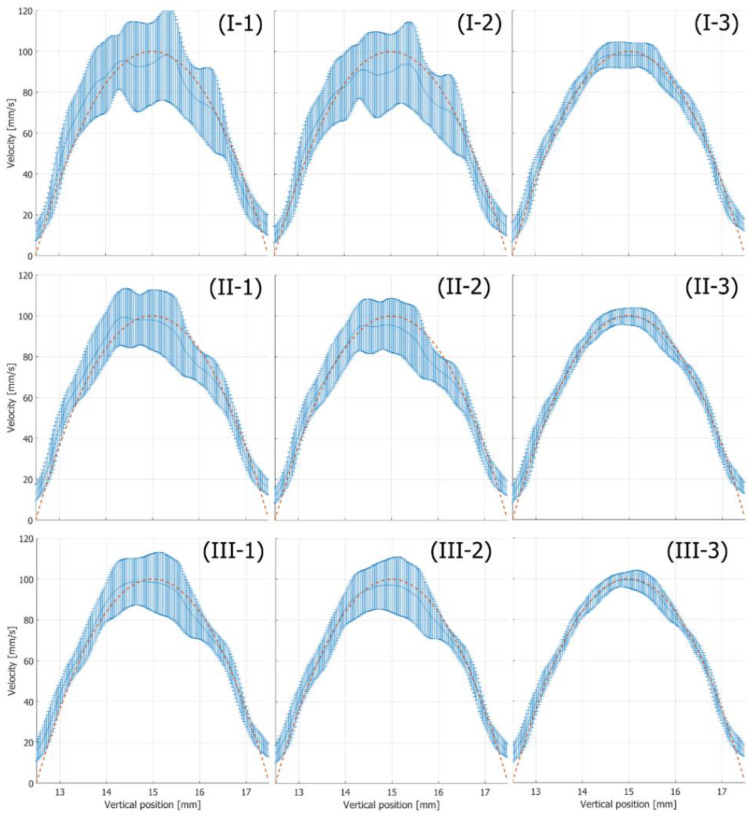
Means and standard deviations of estimated velocity magnitudes (flow tilt angle: 0 degrees, maximum velocity: 100 mm/s). The red dashed line shows the true velocity profile. The plots and vertical bars show means and standard deviations of estimated velocities, respectively. Results were obtained with Method 1 (**I-1**,**II-1**,**III-1**), Method 2 (**I-2**,**II-2**,**III-2**), and Method 3 (**I-3**,**II-3**,**III-3**). Rx beam steering angles were set at 10 (**I-1**–**I-3**), 15 (**II-1**–**II-3**), and 20 (**III-1**–**III-3**) degrees.

In Figure 6, the plots and vertical bars respectively show the means and standard deviations of the velocity magnitudes, which were estimated under flow tilt angle φ and maximum velocity $v_{\max}$ of 0 degrees and 100 mm/s, respectively. The red dashed line shows the true velocity profile. In Figure 6I–III, maximum Rx beam steering angles $\theta_{\max}$ are set at 10, 15, and 20 degrees, respectively, and the estimates in Figure 6(1–3) are obtained with Methods 1, 2, and 3, respectively.

**Figure 7:**
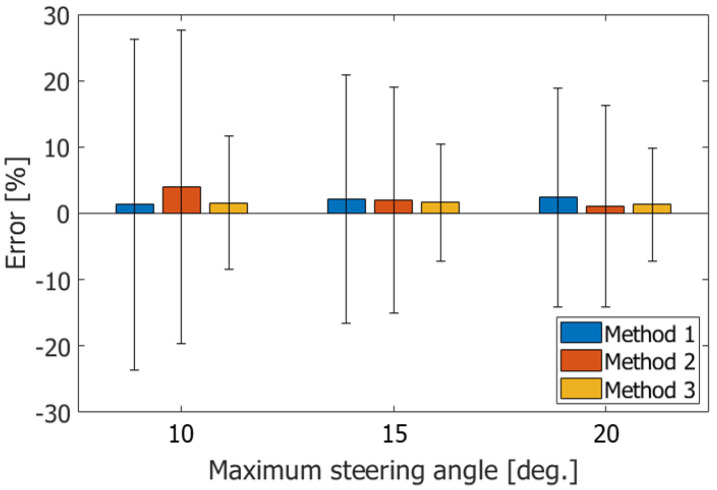
ABEs and RMSEs in estimated velocities obtained with respective methods.

In comparison with the velocity estimates obtained by Method 1 in Figure 6(1), the standard deviations are slightly reduced by estimating the local center frequencies in the beamformed signal using Method 2, as can be seen in Figure 6(2). However, the standard deviations are still large. The standard deviations are reduced significantly using both the center frequency and tilt angle of the wavefront using Method 3, as can be seen in Figure 6(3). Figure 7 summarizes the ABEs and RMSEs obtained by the respective methods. The boxes and vertical bar represent the ABEs and RMSEs, respectively. As can be seen in Figure 7, Method 3 (proposed method) achieved a significantly better performance than Methods 1 and 2. In addition, the proposed method achieved the best performance with an Rx steering angle $\theta_{\max}$ of 20 degrees. Therefore, the proposed method was used with setting $\theta_{\max}$ to 20 degrees in the subsequent examinations.

**Figure 8:**
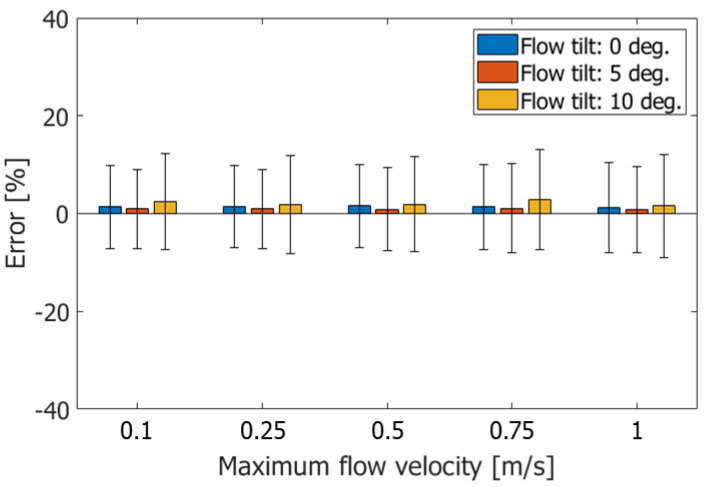
ABEs and RMSEs in estimated velocities obtained with Method 3 at Rx beam steering angle of 20 degrees.

The performance of the proposed method was also evaluated under other conditions. In Figure 8, the ABEs and RMSEs are evaluated at different flow tilt angles φ (0, 5, and 10 degrees) and maximum velocities $v_{\max}$ (100, 250, 500, 750, and 1000 mm/s) are shown by the boxes and vertical bars, respectively. The velocities were estimated with RMSEs of around 10% under different flow tilt angles and maximum flow velocities.

**Figure 9:**
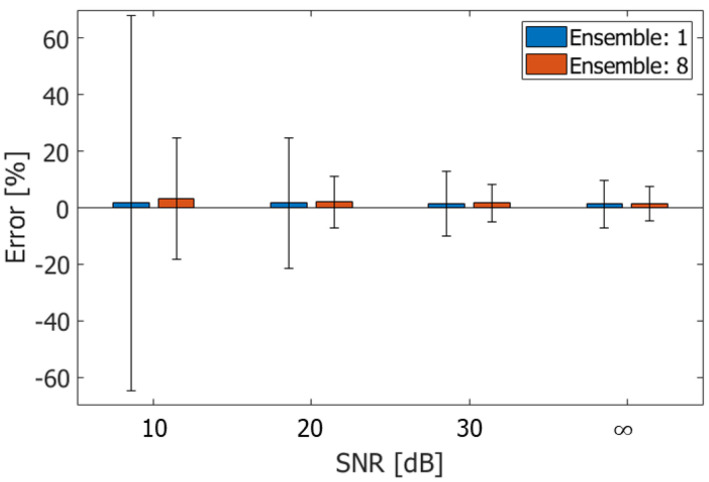
ABEs and RMSEs evaluated by estimating velocities without and with ensemble averaging. The errors are evaluated under different SNRs.

Figure 9 shows the ABEs and RMSEs in the velocities obtained at different signal-to-noise ratios (SNRs). The SNR was altered by adding random noise to the simulated beamformed signal. The flow tilt angle φ and maximum velocity $v_{\max}$ were the same as those in Figure 7. As can be seen in Figure 9, the errors increase with decreasing the SNR. In conventional color flow imaging, ensemble averaging is commonly used to reduce the variance in estimated velocities. The errors were also evaluated with the ensemble averaging of the complex correlation functions for eight frames and shown in Figure 9. As can be seen in Figure 9, the ensemble averaging is effective to reduce the variance in the estimated velocities even in the proposed method.

### 3.2. In Vivo Measurements

As a velocity estimator itself, it was shown by the simulations that the proposed method with focused Tx beams achieved an accuracy in velocity estimation, which was comparable to the method with PW imaging [48]. The feasibility of the proposed method in in vivo imaging and comparison of the methods implemented in FB and PW imaging were investigated by measuring human common carotid arteries in vivo.

**Figure 10:**
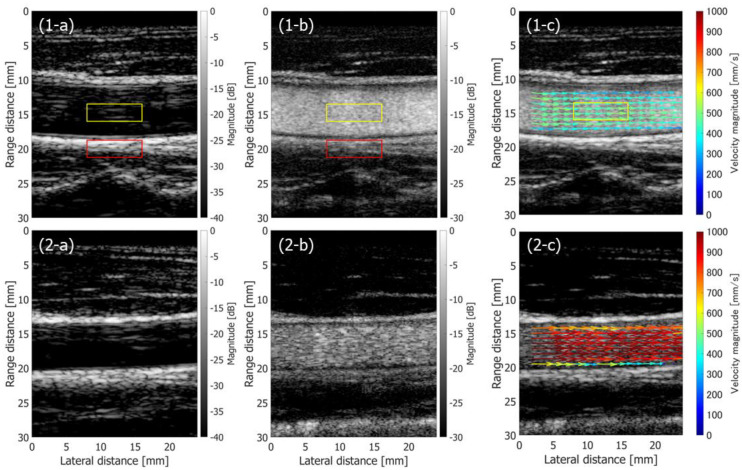
Results on in vivo measurements of 48-year-old (**1-a**,**1-b**,**1-c**) and 26-year-old (**2-a**,**2-b**,**2-c**) healthy subjects obtained with the Tx-Rx sequence developed in this study. (**1-a**,**2-a**) Normal B-mode images. (**1-b**,**2-b**) Clutter-filtered B-mode images. (**1-c**,**2-c**) Flow velocity vectors estimated in cardiac systolic phase are overlaid on blood speckle images. The red and yellow boxes show regions used for evaluation of contrast.

Figure 10(1-a,2-a) show B-mode images of the common carotid arteries of 48-year-old and 26-year-old healthy male subjects, respectively. Figure 10(1-b,2-b) show B-mode images of the 48-year-old and 26-year-old subjects, respectively, obtained from the clutter-filtered beamformed signals.

The clutter-filtered signals were analyzed with Method 3 at Rx maximum beam steering angle $\theta_{\max}$ of 20 degrees. The arrows in Figure 10(1-c,2-c) show the flow velocity vectors in the carotid arteries of the 48-year-old and 26-year-old subjects, respectively, obtained in cardiac systolic phase. The arrows are overlaid on the corresponding blood speckle images. The blood speckle images were obtained by the incoherently summed non-filtered and clutter-filtered beamformed signals. In Figure 10, the complex correlation functions are averaged for eight frames (46.08 ms) before estimating the velocities. As shown in Figure 10(1-c,2-c), systolic flow velocities of around 1000 mm/s are found in the result on the 26-year-old subject, while that of the 48-year-old subject is around 600 mm/s. Physiologically consistent systolic flow velocities and age-related difference in flow velocities are reported in the literature [54], and thus the proposed method is considered feasible in in vivo applications.

**Figure 11:**
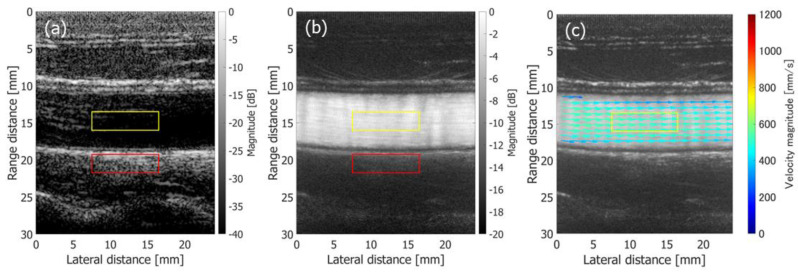
Results on in vivo measurement of a 48-year-old healthy subject obtained with PW imaging [48]. (**a**) Normal B-mode image. (**b**) Clutter-filtered B-mode image. (**c**) Flow velocity vectors estimated in cardiac systolic phase are overlaid on blood speckle image.

Then, the performance of the proposed method in the FB sequence was compared with the method implemented in PW imaging [48]. Figure 11a–c show a normal B-mode image, a clutter-filtered B-mode image, and velocity vectors in cardiac systolic phase overlaid on a blood speckle image obtained from the same 48-year-old subject using the method with PW imaging. The Tx-Rx sequence is described in [48]. The complex correlation functions were averaged for 46.08 ms (80 frames), which was the same as that in Figure 10. It should be noted that the frame rate in the PW imaging was ten times higher than that in the FB imaging.

First the contrast between the vessel lumen and surrounding tissue in the B-mode image (both non-filtered and clutter-filtered) was evaluated. A better contrast in a non-filtered B-mode image would benefit the observation of surrounding tissues including the arterial wall. On the other hand, a better suppression of clutter signals would result in a better contrast in a clutter filtered B-mode image. The contrast between the vessel lumen and surrounding tissue was evaluated as

(20) $$contrast=\left|20\log_{10}\frac{\mu_{L}}{\mu_{T}}\right|\;\left[\mathrm{dB}\right],\;$$

where $\mu_{L}$ and $\mu_{T}$ are the mean echo amplitudes in the regions of interest (ROIs) assigned in the vessel lumen and surrounding tissue, as respectively indicated by the yellow and red rectangles in Figure 10(1-a,1-b) and Figure 11a,b.

**Figure 12:**
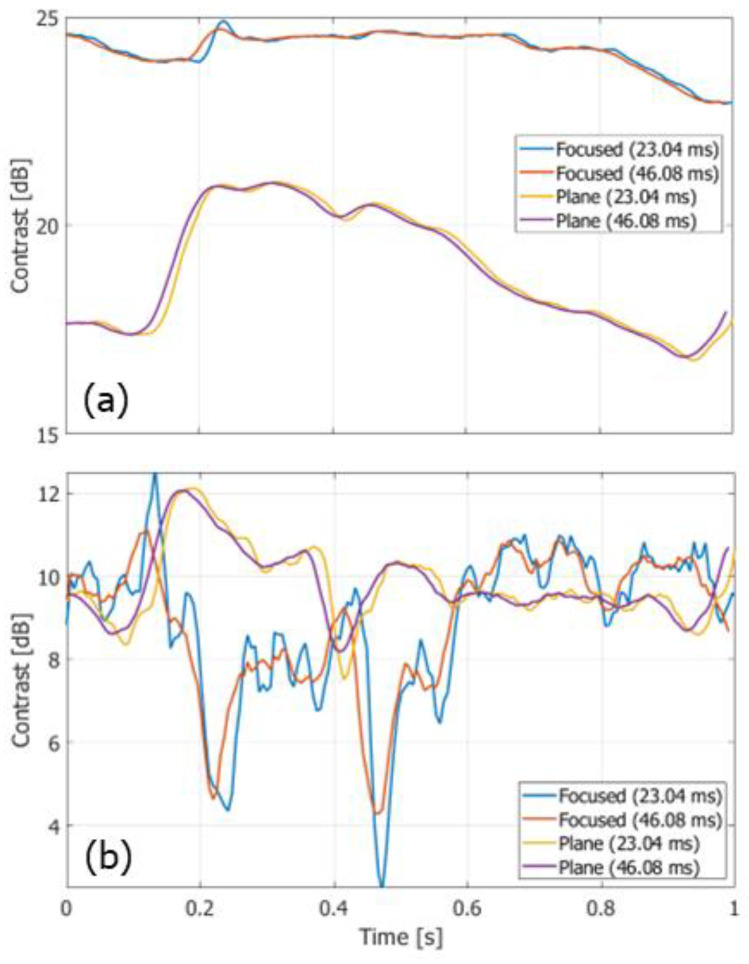
Temporal change in contrast values obtained from non-filtered (**a**) and clutter-filtered (**b**) B-mode images.

Figure 12a,b show the temporal changes in the contrast values obtained from non-filtered and clutter-filtered B-mode images, respectively. Different durations (23.04 ms and 46.08 ms) for averaging the complex correlation functions were examined. The contrast of the non-filtered B-mode image obtained with FB imaging was significantly higher than that obtained with PW imaging. The contrast of the clutter-filtered B-mode image with FB imaging was slightly higher in the slow-flow phase than that with PW imaging, while the contrast with FB imaging was significantly reduced in the fast-flow phase.

**Figure 13:**
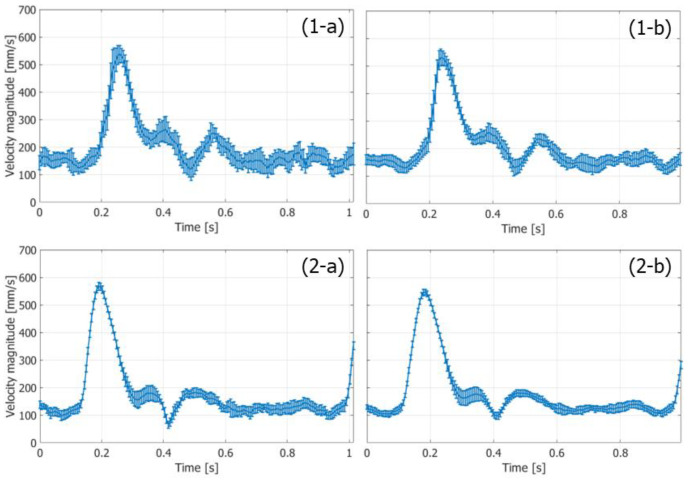
Temporal changes in means (plots) and standard deviations (vertical bars) of magnitudes of velocity vectors at vertical center of ROI. (**1-a**,**1-b**) FB imaging. (**2-a**,**2-b**) PW imaging [48]. (**1-a**,**2-a**) Ensemble length of 23.04 ms. (**1-b**,**2-b**) Ensemble length of 46.04 ms.

**Figure 14:**
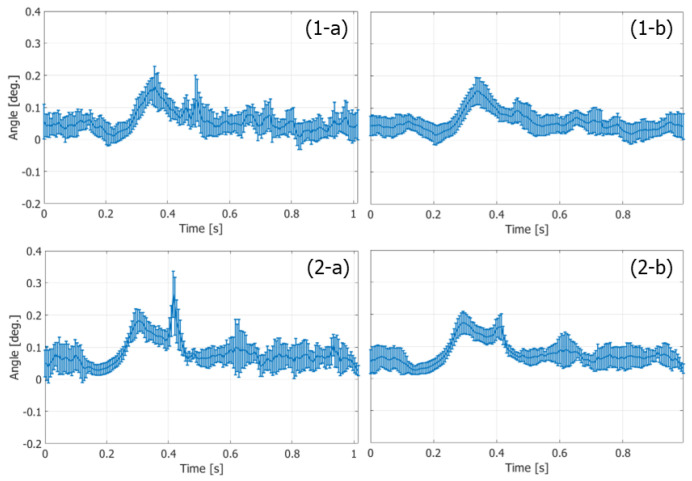
Temporal changes in means (plots) and standard deviations (vertical bars) of angles of velocity vectors at vertical center of ROI. (**1-a**,**1-b**) FB imaging. (**2-a**,**2-b**) PW imaging [48]. (**1-a**,**2-a**) Ensemble length of 23.04 ms. (**1-b**,**2-b**) Ensemble length of 46.04 ms.

**Figure 15:**
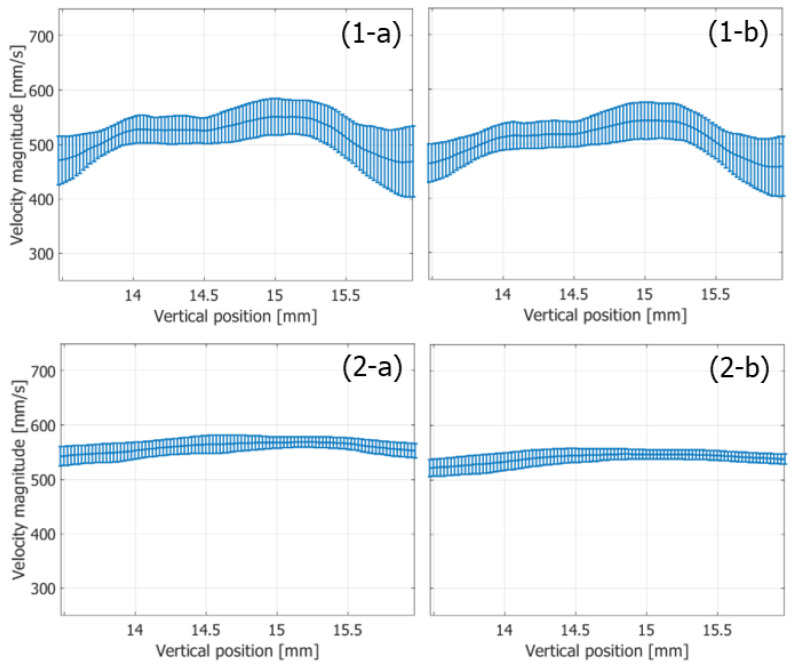
Means (plots) and standard deviations (vertical bars) of magnitudes of velocity vectors along the vertical axis at the time of the maximum mean velocity magnitude. (**1-a**,**1-b**) FB imaging. (**2-a**,**2-b**) PW imaging [48]. (**1-a**,**2-a**) Ensemble length of 23.04 ms. (**1-b**,**2-b**) Ensemble length of 46.04 ms.

**Figure 16:**
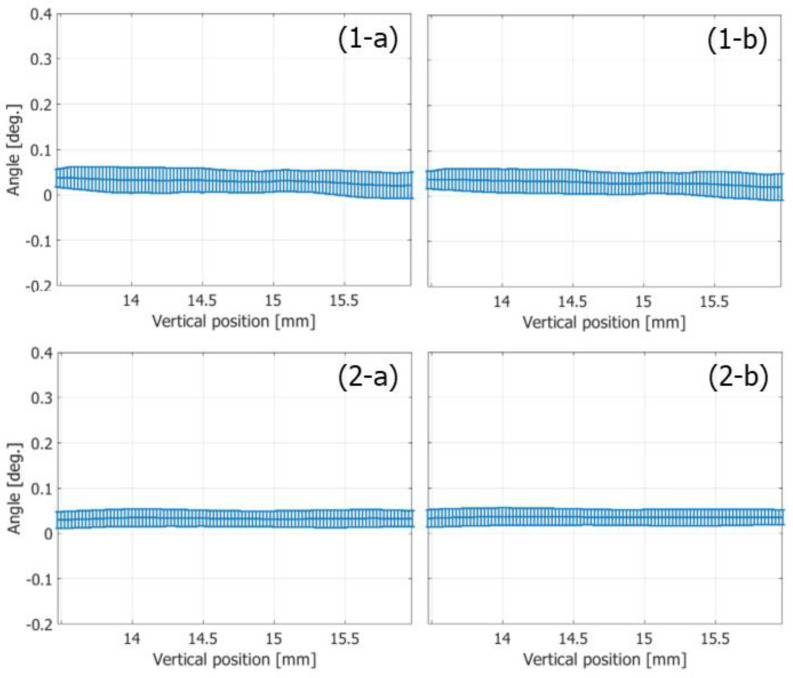
Means (plots) and standard deviations (vertical bars) of angles of velocity vectors along the vertical axis at the time of the maximum mean velocity magnitude. (**1-a**,**1-b**) FB imaging. (**2-a**,**2-b**) PW imaging [48]. (**1-a**,**2-a**) Ensemble length of 23.04 ms. (**1-b**,**2-b**) Ensemble length of 46.04 ms.

The variances of the velocity vectors estimated with FB and PW imaging were also compared. The ROIs were assigned as indicated in Figure 10(1-c) and Figure 11c, where the velocity distributions were relatively homogeneous. The mean and standard deviation in the magnitudes and angles of the velocity vectors were evaluated at each vertical position in the ROI from the velocity vectors estimated in different vertical lines at the corresponding vertical position. Figure 12 and Figure 13 show the temporal changes in the means and standard deviations of the magnitudes and angles at the central vertical position in the ROI, respectively. Figure 14 and Figure 15 show the spatial profiles of the means and standard deviations in the velocity magnitude and angles along the vertical axis, respectively, at the time when the mean velocity magnitude is maximum. In Figure 13, Figure 14, Figure 15 and Figure 16, the means and standard deviations are shown by the plots and vertical bars, respectively.

Since the frame rate in the PW imaging was ten times higher than that in the FB imaging, overall standard deviations in the magnitudes of the estimated velocities were significantly lower in the PW imaging than in the FB imaging, owing to the significantly higher number of frames for averaging the complex correlation functions. However, similar maximum mean velocity magnitudes were obtained with both the FB and PW frameworks. On the other hand, there were not such large differences in the standard deviations in the angles of the velocity vectors estimated with the FB and PW frameworks, and the angle standard deviations were lower than 0.1 degrees.

**Figure 17:**
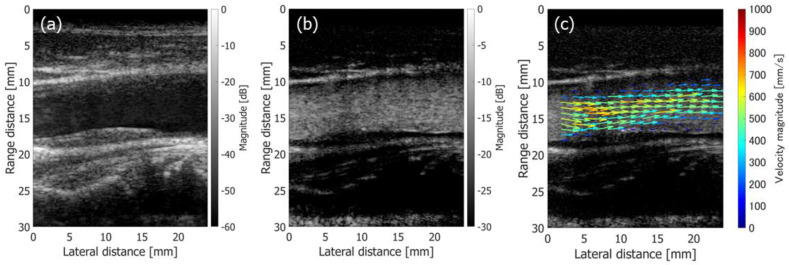
Results on in vivo measurement of a carotid artery with a disturbed flow due to wall thickening. (**a**) Normal B-mode image. (**b**) Clutter-filtered B-mode image. (**c**) Flow velocity vectors estimated in cardiac systolic phase are overlaid on blood speckle image.

Finally, the proposed method was applied to a common carotid artery with a disturbed flow due to thickening of the arterial wall. The results are shown in Figure 17. Figure 17a–c show a normal B-mode image, a clutter-filtered B-mode image, and flow velocity vectors estimated in cardiac systolic phase overlaid on a blood speckle image, respectively. As can be seen in Figure 17c, a reasonable flow velocity distribution is also obtained for a disturbed flow by the proposed method.

## 4. Discussion

This study explored a strategy to implement the two-dimensional wavenumber analysis developed for PW imaging [48] in a multi-line Tx scheme with focused beams. In the focused beam sequence used in the present study, the Tx beam was not steered, and thus the Tx-Rx total steering angle becomes smaller as described in Section 2.3 than that in the case when both the Tx and Rx beams were steered [48]. Since a large Doppler angle increases the variance in estimated velocities [55], it was supposed that the variance was large using our previous method in [41]. Our method for wavenumber analysis reduced the ambiguity in beam steering angles, and the variance in estimated velocities was reduced significantly.

The implementation of the two-dimensional wavenumber analysis was realized by parallel Rx beamforming. Parallel Rx beams are important to estimate the phase difference of the received signals in two neighboring Rx lines, owing to only the tilt of the wavefront because the phase difference would include a component induced by a target motion when the two Rx lines are obtained from different Txs. Therefore, two parallel Rx lines were created in this study. Tilt angle ϕ of a wavefront in a beamformed signal induces a phase difference of $4\pi f_{0}\delta x\sin\phi /c_{0}$. This phase difference is 1.34 radians at Rx line interval δx and wavefront tilt angle ϕ of 0.2 mm and 10 degrees, respectively, when the center frequency $f_{0}$ is 4.8 MHz. This phase difference should be less than π to avoid aliasing. Therefore, the lateral interval between the Rx lines should be narrowed when the proposed method is used with a higher center frequency or larger steering angle.

In this study, two Tx beams were also created in parallel to increase the frame rate. The Tx-Rx sequence requires (30 aperture positions)×(2 Txs per position) =60 Txs to acquire echo signals for one frame. Under such a condition, the frame rate becomes 174 frames per second (fps) when the PRI is 96 ms. This frame rate is far lower than that of 1736 fps in the vector Doppler method with PW imaging [48]. However, the frame rate in the proposed method is still significantly higher than that in conventional color flow imaging. To increase the number of parallel Tx beams is one of the strategies to increase the frame rate in the proposed method. However, as is reported in [40], the cross talks between the parallel Tx beams might be significant when the number of Tx beams is increased.

**Figure 18:**
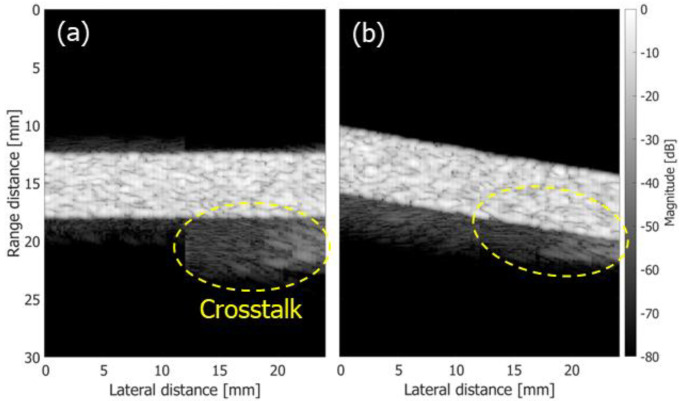
B-mode images obtained from beamformed signals with receiving steering angles of 20 degrees displayed at a dynamic range of 80 dB. (**a**) φ = 0 degrees. (**b**) φ = 10 degrees.

Although only two Tx beams were created in this study, the crosstalk between the two Tx beams affected the velocity estimation. In Figure 8, the errors in the estimated velocity vectors at flow tilt angle φ of 10 degrees are slightly larger than those at 0 and 5 degrees. To consider the reason for this phenomenon, B-mode images of the simulation phantoms at flow tilt angles φ of 0 and 10 degrees are shown in Figure 18a,b, respectively. The Rx beam steering angles were set at −20 degrees in both cases. It should be noted that the B-mode images are displayed with a large dynamic range of 80 dB. In Figure 18a, the crosstalk artifact is found beneath the simulation phantom. In Figure 18b, such an artifact is considered overlapping with the simulation phantom due to the tilt of the phantom and affected the velocity estimation.

Even if the frame rate in the vector Doppler method with a focused beam strategy could be increased further, the high-frame-rate imaging with unfocused Tx beams such as a plane wave would still achieve a significantly higher frame rate. Such a high frame rate is considered beneficial for blood flow imaging. In Figure 12b, the contrast in the clutter-filtered B-mode image obtained with PW imaging is significantly higher in the fast-flow phase than that with FB imaging. In SVD clutter filtering, the temporal intervals between the datasets were 5.76 and 0.576 μs in the FB and PW frameworks, respectively. Due to the large temporal interval in the FB imaging, a significant part of signals might behave as echoes from slowly moving targets due to the aliasing phenomenon and removed by clutter filtering. At the dicrotic notch (around 0.5 s), another significant decrease occurs in the contrast obtained with the FB framework because the difference between velocities of the blood flow and arterial wall becomes smaller due to an increase in the motion velocity of the arterial wall and a decrease in the blood flow velocity. On the other hand, although there were some fluctuations, consistent contrast values were obtained by PW imaging throughout a cardiac cycle. In addition, as shown in Figure 13 and Figure 15, the PW framework achieved significant smaller standard deviations in velocity magnitudes than the FB framework. These facts prove the significant benefits of PW imaging in blood flow measurements.

The benefit of the proposed method with FB imaging is the high contrast between vessel lumen and surrounding tissue in normal B-mode imaging. Such a benefit might be further enhanced by implementing tissue harmonic imaging in the proposed method with FB imaging because tissue harmonic imaging is difficult in PW imaging due to the inherently lower sound pressure than in FB imaging. A strategy to implement the pulse inversion technique, which is an effective method for extracting harmonic components, should be explored to realize tissue harmonic imaging in the proposed method.

As described above, both FB and PW imaging have benefits and shortcomings. The implementation of the vector Doppler method in FB imaging presented in this paper adds a new choice for simultaneous observation of blood flow and surrounding tissue. On the other hand, PW imaging has predominant properties for blood flow imaging. Thus, a time division sequence including both FB emissions for tissue imaging and PW emissions for blood flow imaging is another possibility for optimal imaging of vascular dynamics. In such a time division sequence, the number of emissions available for clutter filtering should be limited, and clutter filtering in such a time division sequence might be one of the topics to be investigated. Development of an effective clutter filter with a small number of emissions would also be beneficial for the proposed FB framework to enable real-time imaging. Since the beamformed signals for approximately 1 s were used for clutter filtering with SVD, the proposed FB framework described in this paper cannot be performed in real time. An effective clutter filter, which works well with a smaller number of frames, would be required for real-time imaging.

Cardiac blood flow imaging is also important for the evaluation of heart function. The multi-line acquisition with FBs was used for myocardial motion imaging [40]. Clutter filtering with SVD is commonly used for vascular flow imaging [53] and is not common in cardiac flow imaging where more clutter motion is present. Although SVD was shown to be effective for clutter filtering in high-frame-rate cardiac flow imaging with diverging wave emissions [56], the feasibility in multi-line acquisition with a significantly lower frame rate has not been demonstrated. Further investigations are necessary to apply the proposed FB framework to cardiac flow imaging.

## 5. Conclusions

In this study, the two-dimensional wavenumber analysis was implemented in a multi-line Tx sequence with focused beams to improve the velocity estimates in the vector Doppler method. It was shown by the simulations that the proposed method could estimate the velocity vectors with RMSEs of approximately 10%, and the in vivo experimental results showed physiologically consistent velocity distributions in the two healthy subjects. The proposed method realized vector flow mapping at a frame rate of 174 fps, which is significantly higher than that in conventional color flow imaging. Such a method would be beneficial for detailed analyses of blood flow dynamics. The effectiveness of the proposed method in more complexly diseased arteries will be investigated in our future work.

## Figures and Tables

**Table 1:** Parameters in simulations and in vivo measurements.

Parameters	Value
Tx center Frequency	4.8 MHz
Element pitch	0.2 mm
PRF	10 kHz (simulation) 10.417 kHz (in vivo)
Sampling frequency	31.25 MHz
Rx F-number	2.34
Correlation kernel size	*x*: 1.0 mm, *z*: 0.825 mm
Ensemble size	1–8 (simulation), 4, 8 (in vivo)

## Data Availability

Not applicable.
